# Landscape of Molecular Events in Pituitary Apoplexy

**DOI:** 10.3389/fendo.2018.00107

**Published:** 2018-03-20

**Authors:** Prakamya Gupta, Pinaki Dutta

**Affiliations:** ^1^Department of Neurosurgery, Postgraduate Institute of Medical Education and Research, Chandigarh, India; ^2^Department of Endocrinology, Postgraduate Institute of Medical Education and Research, Chandigarh, India

**Keywords:** cytokines, growth factors, hypoxia-inducing factor, matrix metalloproteinase-2/9, pituitary apoplexy, tumor necrosis factor-α, vascular endothelial growth factor

## Abstract

Apoplectic pituitary adenomas cause significant morbidity and even mortality. The pituitary apoplexy denotes a pituitary adenoma presenting with hemorrhage and/or infarction, implementation in remedial effects of various of drugs in pituitary apoplexy is a promising pharmacogenomic field in the near future adenoma treatment. Indisputably, this is an important horizon for complicated pituitary adenomas. In a pituitary adenoma, the interplay between genetic, cytokine, and growth factors promotes the pathogenic transformation into an apoplectic formation. However, till date, little is known about how all these factors together lead to the pathogenesis of apoplectic pituitary. The vascular endothelial growth factor, tumor necrosis factor-α (TNF-α), pituitary tumor-transforming gene (PTTG), matrix metalloproteinase-2/9 (MMP-2/9), proliferating marker (Ki-67), as well as hypoxia-inducing factor are the major contributing factors involved in pituitary apoplexy. The molecular mechanism involved in pituitary apoplexy has never been described so far. In this review, we discuss the various proteins/cytokines/growth factors and signaling molecules which are involved in the pathogenesis of pituitary apoplexy and their potential role as biomarkers or as therapeutic targets.

## Introduction

Pituitary apoplexy occurs due to hemorrhage or ischemic infarction of the pituitary gland. It is often associated with the sudden-onset of a headache, visual deterioration, vomiting, altered sensorium, ophthalmoplegia, fever, signs of meningeal irritation, and sometimes with pituitary hormones deficiency (1, 2). The life-threatening condition possesses significantly high morbidity and even mortality. The apoplectic tumor exhibits a wide range of clinical behavior: some are small, non-invasive and some are giant and aggressive, infiltrating into the cavernous sinus. The non-functioning pituitary adenomas are most commonly associated with pituitary apoplexy (3). Moreover, silent corticotroph adenomas are prone to hemorrhage than any other subtype (3). In an adenomatous pituitary, it is postulated that the growing tumor outstrips its blood supply causing ischemic necrosis. However, it is difficult to explain the mechanism in a non-adenomatous pituitary. Henceforth, it is speculated that the involvement of extrinsic factors, such as medication and/or systemic diseases, might also trigger changes leading to necrosis or hemorrhage of the pituitary. Till date, several predisposing factors were described, however, the common precipitating factors in the majority of patients include: medications (Antithrombotic therapy, Dopamine agonists), associated medical conditions (Diabetes mellitus, Arterial hypertension), surgery (Cardiac surgery), head trauma, endocrinological testing [GHRH, TRH, corticotropin releasing hormone (CRH) stimulation test], anticoagulants, and/or snakebite (4, 5).

In this review, we discuss how these precipitating factors triggering the downstream molecular pathways which are responsible for the development of pituitary apoplexy. Despite emerging evidences, there is scanty literature about how these precipitating factors contribute to haemorrhagic and/or infarction of the pituitary adenoma. Although many underlying molecular events in an apoplectic pituitary were discovered, a reliable prognostic marker still remains to be identified. The vascular endothelial growth factor (VEGF), tumor necrosis factor-α (TNF-α), HIF-1α (hypoxia-inducing factor-1 α), pituitary tumor-transforming gene (PTTG), and matrix metalloproteinase-2/9 (MMP-2/9) currently represent promising candidates with the potential to guide the management of patients with apoplectic pituitary. Knowing more about these molecular pathways might help to identifying the susceptible genetic variants and facilitate the development of medications to prevent the formation in a known case or obliterate a formed apoplectic adenoma.

## Key Factors Involved in Pituitary Apoplexy and the Pathways Involved

### Vascular Endothelial Growth Factor

Angiogenesis is a complex process that is tightly regulated by pro-angiogenic and anti-angiogenic factors. These factors communicate *via* autocrine and paracrine signaling. Until now, several studies have shown a strong correlation between intratumoral hemorrhage and VEGF overexpression (6, 7). Though VEGF is expressed in both normal and adenomatous pituitary, some reported higher VEGF expression in normal pituitary glands compared to adenomatous pituitary, while the reverse has also been published (8). Moreover, a third report showed no significant difference in VEGF immunostaining between normal and adenomatous pituitary glands (9). A study by Lee et al. has even shown that plasma VEGF levels are significantly elevated as compared to healthy controls and decreased within 1 month after stereotactic radiosurgery (10). VEGF, a homodimeric mitogenic glycoprotein, is the most potent inducer of angiogenesis, vasculogenesis, and vascular permeability. Although the human VEGF is encoded by a single gene, VEGF exists in four different isoforms (121, 165, 189, 206 kDa). VEGF-A is the best characterized and commonly referred as VEGF (11). This 21- to 46-kDa protein was reported to be responsible for intratumoral hemorrhage of pituitary adenomas (6, 12). The activated VEGF triggers a broad spectrum of signaling cascades such as the PI3K/AKT pathway. The stimulated VEGF promotes endothelial cell survival, proliferation, and angiogenesis, thereby predisposing for haemorrhagic events (13–15). New drugs such as temozolomide and anti-VEGF monoclonal antibody play an important role in the management of aggressive pituitary adenoma (16–18). However, the anti-VEGF therapy may act as a supplementary therapy for conservative management of pituitary apoplexy.

The biological activities of VEGFs are mediated by two unique tyrosine kinase receptors: VEGFR1 [or fms-like tyrosine kinase] and VEGFR2 [or fetal liver kinase 1/kinase insert domain-containing receptor (Flk-1/KDR)]. These two receptors are involved in angiogenesis and signal transduction pathways. The Flk-1/KDR (VEGFR2) is now believed to specifically bind to VEGF in vascular endothelial cells. The Neuropilin 1, another neuronal receptor that mediates the repulsive growth cone guidance, was recently shown *in vitro* to function in endothelial cells as an isoform-specific VEGF receptor as well as a VEGF receptor 2 co-receptor. The microvessel density represents a measure of angiogenesis and may be used as an indicator of neoplastic aggressiveness. The growing and metastatic solid neoplasms develop high microvascular density (19). However, in case of pituitary adenomas, lower vascularity is observed as compared to normal pituitary tissue, thus suggesting role of angiogenic inhibitors in the pathologic processes associated with these lesions (20, 21). It may be speculated that the lower angiogenesis may, therefore, contribute to the slow pace of growth characteristic of most pituitary adenomas and explain the relative rarity of metastases. The microvascular density of apoplectic pituitary assessed using different vascular endothelial markers, including platelet endothelial cell adhesion molecule (CD31) and endoglin (CD105) showed a strong correlation with the VEGF expression in apoplectic pituitary. However, no association was observed with apoplectic pituitary adenomas (22). Despite extensive research on angiogenesis, the exact relationship between angiogenesis, microvascular density, tumor bleed, or infarction and the clinical behavior of pituitary adenomas still remain undeciphered.

**Figure 1:**
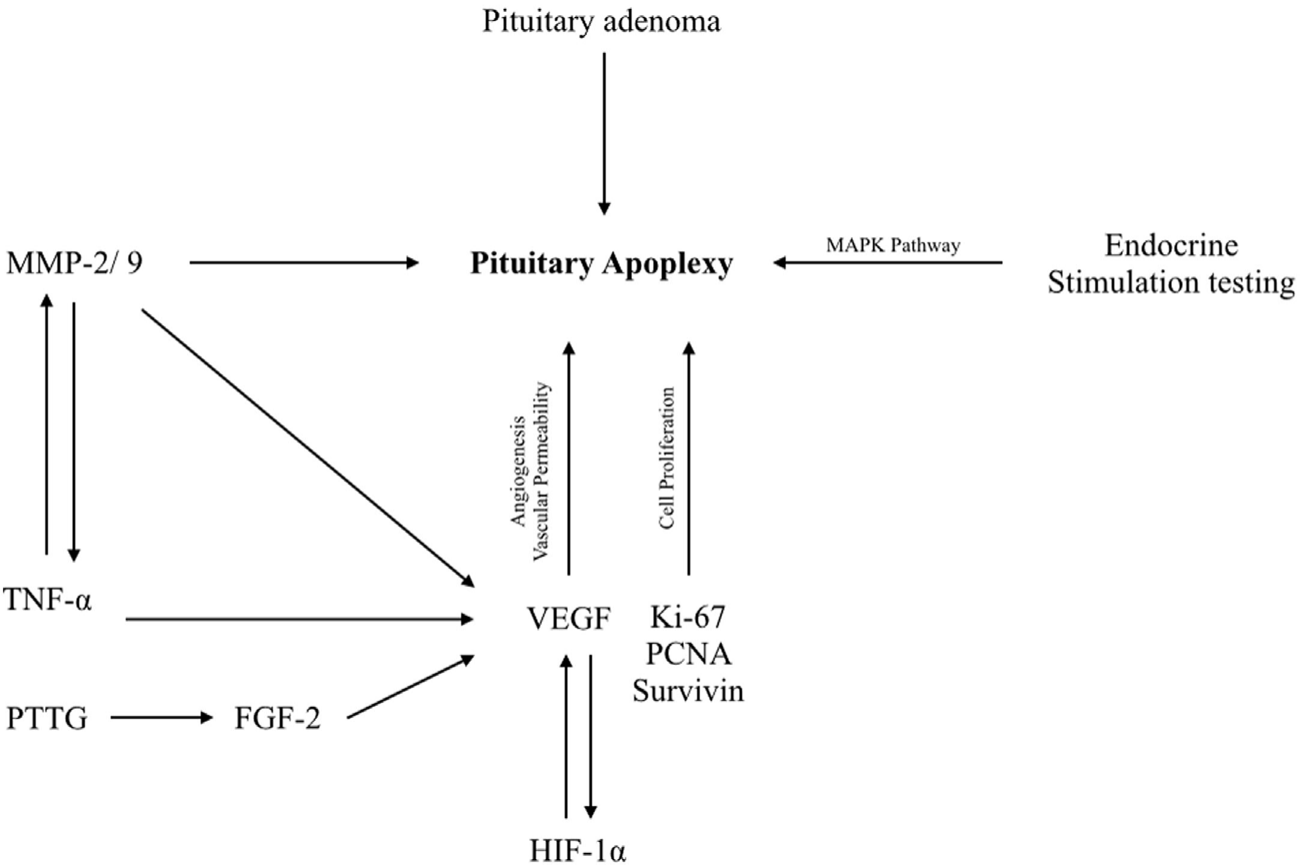
Schematic diagram showing molecular events in pituitary apoplexy. HIF-1α, hypoxia-inducing factor-1α; Ki-67, proliferation marker; MAPKs, mitogen-activated protein kinases; MMP-2/9, matrix metalloproteinase-2/9; TNF- α, tumor necrosis factor-alpha; VEGF, vascular endothelial growth factor.

The increased vascular permeability is possibly induced by VEGF overexpression which may results in fluid exudation and/or cyst formation. This may lead to surge in the tissue pressure in the adenoma (23, 24). The pituitary adenomas are partially irrigated through the pituitary portal system. Thus, even a mild increase in tissue pressure within the adenomas may suffice to overwhelm the low perfusion pressure and results in pituitary adenoma tissue necrosis and thus intratumoral hemorrhage. The proteins/genes/growth factors that participate in pituitary apoplexy are enlisted in Table 1 and Figure 1.

**Table 1:** Summary of proteins/genes/cytokines/growth factors that participate in pituitary apoplexy.

Pathway	Role in pituitary apoplexy	Reference
VEGF	Tumor angiogenesis	(6–8, 12)
Endoglin (CD105, CD31)	Microvascular density	(22)
PTTG and FGF	Pituitary tumorigenesis and growth	(14, 27, 28)
Ki-67	Cell proliferation	(29, 31–34)
TNF-α	Angiogenesis, vascular hyperpermeability, and destruction of vascular integrity	(7)
HIF-1α	Hypoxia, activates VEGF	(37, 38)
MMP-2/9	Degradation of extracellular matrix, vascular permeability	(7, 43, 46, 47)

*FGF, fibroblast growth factor; HIF-1α, hypoxia-inducing factor-1α; MMP-2/9, matrix metalloproteinase-2/9; PTTG, pituitary tumor-transforming gene; Ki-67, proliferation marker; TNF- α, tumor necrosis factor-alpha; VEGF, vascular endothelial growth factor*.

### Pituitary Tumor-Transforming Gene

The PTTG is located on chromosome 5q33 and is abundantly expressed in most human pituitary tumors (25). PTTG plays an important role in pituitary cell transformation and tumor formation (26). The PTTG is abundantly expressed in pituitary adenomas and various other tumors types and is known to stimulate fibroblast growth factor-2 (FGF-2) (27). The activated FGF-2 further regulates endothelial expression of VEGF. Thus, FGF-2 is involved in the angiogenesis and thus the development of pituitary tumors through autocrine and paracrine mechanisms. The tumor cell proliferation then regulates the expression of PTTG (14, 28).

### Ki-67

According to the recent WHO classification, Ki-67 or MIB-1 is one of the most reliable and widely used cell proliferation marker (29, 30). A number of studies have found correlation between higher Ki-67 values and invasion, aggressiveness, and recurrence of pituitary adenomas (29, 31). Despite extensive research of Ki-67 on pituitary adenomas, there are only a few studies on Ki-67 labeling index in pituitary apoplexy, and the findings have been inconsistent. The expression pattern differed between the pituitary adenoma subtypes. Ki-67 is significantly high in invasive, aggressive pituitary adenomas as compared to non apoplectic adenomas (32–34). Moreover, a study by Cinar et al. found that Ki-67 labeling index, p53 positivity, antithrombotic therapy, and somatostatin analog do not predispose to pituitary apoplexy (3). Emerging studies using deoxyribonucleic acid and microribonucleic acid microarray analyses will aid researchers in gaining a deeper understanding of exact mechanism of Ki-67 induced pituitary apoplexy.

### Inflammation and TNF-α

Tumor necrosis factor-α is a well-known cytokine which is involved in multiple pathological processes such as inflammation, angiogenesis, vascular hyperpermeability, and destruction of vascular integrity. TNF-α is amplifying several signaling pathways leading to inflammation or apoptosis/survival by recruiting different adaptor proteins. In the study by Xiao et al. using mice model of pituitary tumor cell xenografts, it was observed that TNF-α promoted haemorrhagic transformation of the pituitary *via* upregulating VEGF and MMP-9 (7). However, it is not clear if TNF-α acts alone or in combination with other risk factors, environmental exposures, or underlying disease conditions. The study has also demonstrated that administration of both VEGF and MMP-9 inhibitors attenuated but not abrogated TNF-α mediated hemorrhagic transformation. The inability of VEGF inhibitors averting the TNF-α mediated MMP-9 expression excludes the possibility of direct effects of VEGF on MMP-9 expression. Thus suggesting, other targets may also contribute to hemorrhagic transformation, besides VEGF and MMP-9. Apart from TNF-α, other mechanisms might be involved in haemorrhagic transformation of pituitary adenomas. However, further investigations are required to validate the results.

### Hypoxia-Inducing Factor-1α (HIF-1α)

Decreased oxygen supply is a critical factor in the angiogenesis of tumor growth. The hypoxia-inducing factors (HIF) are transcription factors that respond to changes in available oxygen in cellular environment, especially to decrease in oxygen or hypoxia (35). The HIF expression is downregulated *via* proteosomal degradation (36). However, in an advent of reduced oxygen supply, the proteasome-mediated degradation of HIF is blocked thus increasing the HIF expression. The HIF-1 heterodimer binds to the hypoxia response element, thereby activating VEGF. The recently cloned small RWD-domain containing protein sumoylation enhancer (RSUME) was shown to increase protein levels of HIF-1α. Indeed, RSUME plays an important role in initiating pituitary tumor neovascularization through regulating HIF-1α levels and subsequently VEGF-A production and may, therefore, be involved in pituitary adenoma progression (37, 38). A recent study by Li et al. has shown that the tyrosine kinase receptor discoidin domain receptor l (DDR1) is overexpressed in hypoxic pituitary adenoma. The overexpressed DDR1 protein further increased the expression of MMP-2/9 which caused haemorrhagic/infarction of the pituitary adenoma. From the above studies, it can be deduced that the fast growing tumor cells tend to outnumber their blood supply, thereby predisposing themselves to sublethal hypoxia which in turn activates HIF-1α activity leading to hemorrhagic transformation in pituitary adenomas. Thus, the HIF-1 or its downstream signaling molecules may be an useful adjunct in the clinical management of pituitary tumors (39).

### Matrix Metalloproteinase-2/9 (MMP-2/9)

Matrix metalloproteinase (MMPs) are proteolytic enzymes involved in the degradation of extracellular matrix thus causing vascular permeability. MMP-9, a member of the MMP protein family, is associated with intracerebral hemorrhage such as aneurysmal diseases (40–42). Moreover, elevated expression of MMP-9 is observed in haemorrhagic pituitary adenomas as compared to non-haemorrhagic tumors (43). These findings suggest that an overexpression of either VEGF or MMP-9 can cause instability of the vessels, thereby predisposing to haemorrhagic events. A recent study by Chen et al. has shown an important role of MMP-2/9 in the development of pituitary adenoma and the mechanism of immune escape in pituitary adenoma. They showed that activated NF-κB upregulates the expression of MHC class I polypeptide-related sequence A (MICA) and thereby induces the expression of MMP-9 (44). This could be used as a new target for inhibiting tumor cell immune escape. A study by Paez Pereda et al. showed that the tumor cells secrete MMPs which in turn control the hormone secretion and cell proliferation (45). The MMP-9 and tissue inhibitor of matrix metalloproteinase-2 (TIMP) are potential prognostic biological markers in invasive prolactinomas (46). In addition to VEGF, TNF-α also regulates the functioning of MMP-9 and thus has been implicated in hemorrhagic transformation (47). Moreover, high levels of MMP-9 are observed in hemorrhagic pituitary adenomas (43), thereby suggesting a possible role of MMP-9 in the development of hemorrhage within the pituitary adenomas. A study by Xiao et al. demonstrated that TNF-α upregulates the VEGF and MMP-9 in prolactin secreting rat cell line (MMQ cells) (7). These observations are consistent with previous studies about TNF-α function in other human cells (48–50).

## Mitogen-Activated Protein Kinases (MAPKs)

Mitogen-activated protein kinases are a family of intracellular signaling proteins consisting of JNKs, p38 MAPKs, and extracellular signal-regulated kinases (ERKs). The MAPK pathway members can be stimulated in the pituitary stimulation testing by CRH, gonadotropin releasing hormone, or insulin. The activated MAPKs are reported to induce VEGF, thereby promoting neovascularization and proliferation in glioblastoma cells (51). Pituitary apoplexy during endocrinological testing can be attributed to be *via* this pathway.

## Non-Contributory Factors

Other factors such as age, gender, p53 positivity, and other co-morbidities such as diabetes mellitus, hypertension, use of somatostatin analogs, and anticoagulant use were shown to be non-contributory to the predisposition of pituitary apoplexy (3).

## Limitations and Way Forward

Pituitary apoplexy is currently defined as a clinical symptom that can be confirmed radiologically or pathologically. Due to scanty literature in the field, this review proposes the possible underlying pathways responsible for pituitary apoplexy in terms of analyzing a bunch of molecular targets, i.e., VEGF, TNF-α, HIF-1α, MMP-2/9, PTTG, and Ki-67. The analysis of possible constituents of pituitary apoplexy pathways is seemingly relative not encircling the long list of predefined molecules. However, including basic molecular studies of cell damage, namely intrinsic (cellular suicide, e.g., apoptosis) and extrinsic (external threat from surrounding) pathways will help in deciphering the exact mechanism of pituitary apoplexy. Moreover, unknown genes, cytokines, proteins might be involved in two major phenomena such as hemorrhage and infarction by being most effective pathogenetic mechanisms of pituitary apoplexy.

Despite extensive research on pituitary adenoma, there is scanty literature on the etio-pathogenesis of apoplectic pituitary adenoma. Genomics, proteomic, and metabolomics study on large sample sizes are needed to better understand the mechanism and thus may help in the management of patients with pituitary apoplexy.

## Tribute

This paper is dedicated to our mentor/friend/senior professor Late Prof. K. K. Mukherjee, Department of Neurosurgery, Postgraduate Institute of Medical Education and Research, Chandigarh, India, who always inspired and encouraged us to work on pituitary adenoma and see a holistic view.

## Author Contributions

All authors contributed equally.

## Conflict of Interest Statement

The authors declare that the research was conducted in the absence of any commercial or financial relationships that could be construed as a potential conflict of interest.

## References

[1] Rajasekaran S Vanderpump M Baldeweg S Drake W Reddy N Lanyon M UK guidelines for the management of pituitary apoplexy. Clin Endocrinol (2011) 74:9–20. 10.1111/j.1365-2265.2010.03913.x 21044119

[2] Baldeweg SE Vanderpump M Drake W Reddy N Markey A Plant GT Society for Endocrinology Endocrine Emergency guidance: emergency management of pituitary apoplexy in adult patients. Endocr Connect (2016) 5:G12–5. 10.1530/EC-16-0057 27935817 PMC5314810

[3] Cinar N Tekinel Y Dagdelen S Oruckaptan H Soylemezoglu F Erbas T Cavernous sinus invasion might be a risk factor for apoplexy. Pituitary (2013) 16:483–9. 10.1007/s11102-012-0444-2 23179962

[4] Semple PL Jane JA Jr Laws ER Jr. Clinical relevance of precipitating factors in pituitary apoplexy. Neurosurgery (2007) 61:956–61. 10.1227/01.neu.0000303191.57178.2a 18091272

[5] Chang CV Araujo RV Nunes VS Cirqueira CS Felicio AC Predisposing factors for pituitary apoplexy. In: Turgut M Mahapatra AK Powell M Muthukumar N, editors. Pituitary Apoplexy. Berlin, Heidelberg: Springer (2014). p. 21–4.

[6] Arita K Kurisu K Tominaga A Sugiyama K Eguchi K Hama S Relationship between intratumoral hemorrhage and overexpression of vascular endothelial growth factor (VEGF) in pituitary adenoma. Hiroshima J Med Sci (2004) 53:23–7. 15453394

[7] Xiao Z Liu Q Mao F Wu J Lei T TNF-alpha-induced VEGF and MMP-9 expression promotes hemorrhagic transformation in pituitary adenomas. Int J Mol Sci (2011) 12:4165–79. 10.3390/ijms12064165 21747731 PMC3131615

[8] Lloyd RV Scheithauer BW Kuroki T Vidal S Kovacs K Stefaneanu L Vascular endothelial growth factor (VEGF) expression in human pituitary adenomas and carcinomas. Endocr Pathol (1999) 10:229–35. 12114703 10.1007/BF02738884

[9] Viacava P Gasperi M Acerbi G Manetti L Cecconi E Bonadio AG Microvascular density and vascular endothelial growth factor expression in normal pituitary tissue and pituitary adenomas. J Endocrinol Invest (2003) 26:23–8. 10.1007/BF03345118 12602530

[10] Lee KM Park SH Park KS Hwang JH Hwang SK Analysis of circulating endostatin and vascular endothelial growth factor in patients with pituitary adenoma treated by stereotactic radiosurgery: a preliminary study. Brain Tumor Res Treat (2015) 3:89–94. 10.14791/btrt.2015.3.2.89 26605263 PMC4656901

[11] Moreira IS Fernandes PA Ramos MJ Vascular endothelial growth factor (VEGF) inhibition – a critical review. Anticancer Agents Med Chem (2007) 7:223–45. 10.2174/187152007780058687 17348829

[12] Jin Kim Y Hyun Kim C Hwan Cheong J Min Kim J Relationship between expression of vascular endothelial growth factor and intratumoral hemorrhage in human pituitary adenomas. Tumori (2011) 97:639–46. 10.1700/989.10725 22158497

[13] Gerber HP McMurtrey A Kowalski J Yan M Keyt BA Dixit V Vascular endothelial growth factor regulates endothelial cell survival through the phosphatidylinositol 3’-kinase/Akt signal transduction pathway. Requirement for Flk-1/KDR activation. J Biol Chem (1998) 273:30336–43. 9804796 10.1074/jbc.273.46.30336

[14] McCabe CJ Boelaert K Tannahill LA Heaney AP Stratford AL Khaira JS Vascular endothelial growth factor, its receptor KDR/Flk-1, and pituitary tumor transforming gene in pituitary tumors. J Clin Endocrinol Metab (2002) 87:4238–44. 10.1210/jc.2002-020309 12213878

[15] Hoeben A Landuyt B Highley MS Wildiers H Van Oosterom AT De Bruijn EA Vascular endothelial growth factor and angiogenesis. Pharmacol Rev (2004) 56:549–80. 10.1124/pr.56.4.3 15602010

[16] Korsisaari N Ross J Wu X Kowanetz M Pal N Hall L Blocking vascular endothelial growth factor-A inhibits the growth of pituitary adenomas and lowers serum prolactin level in a mouse model of multiple endocrine neoplasia type 1. Clin Cancer Res (2008) 14:249–58. 10.1158/1078-0432.CCR-07-1552 18172277

[17] Luque GM Perez-Millan MI Ornstein AM Cristina C Becu-Villalobos D Inhibitory effects of antivascular endothelial growth factor strategies in experimental dopamine-resistant prolactinomas. J Pharmacol Exp Ther (2011) 337:766–74. 10.1124/jpet.110.177790 21406548

[18] Di Ieva A Rotondo F Syro LV Cusimano MD Kovacs K Aggressive pituitary adenomas – diagnosis and emerging treatments. Nat Rev Endocrinol (2014) 10:423–35. 10.1038/nrendo.2014.64 24821329

[19] Liotta LA Steeg PS Stetler-Stevenson WG Cancer metastasis and angiogenesis: an imbalance of positive and negative regulation. Cell (1991) 64:327–36. 1703045 10.1016/0092-8674(91)90642-c

[20] Turner HE Nagy Z Gatter KC Esiri MM Harris AL Wass JA Angiogenesis in pituitary adenomas and the normal pituitary gland. J Clin Endocrinol Metab (2000) 85:1159–62. 10.1210/jcem.85.3.6485 10720055

[21] Turner HE Harris AL Melmed S Wass JA Angiogenesis in endocrine tumors. Endocr Rev (2003) 24:600–32. 10.1210/er.2002-0008 14570746

[22] Lee JS Park YS Kwon JT Nam TK Lee TJ Kim JK Radiological apoplexy and its correlation with acute clinical presentation, angiogenesis and tumor microvascular density in pituitary adenomas. J Korean Neurosurg Soc (2011) 50:281–7. 10.3340/jkns.2011.50.4.281 22200007 PMC3243828

[23] Arafah BM Prunty D Ybarra J Hlavin ML Selman WR The dominant role of increased intrasellar pressure in the pathogenesis of hypopituitarism, hyperprolactinemia, and headaches in patients with pituitary adenomas. J Clin Endocrinol Metab (2000) 85:1789–93. 10.1210/jcem.85.5.6611 10843153

[24] Zayour DH Selman WR Arafah BM Extreme elevation of intrasellar pressure in patients with pituitary tumor apoplexy: relation to pituitary function. J Clin Endocrinol Metab (2004) 89:5649–54. 10.1210/jc.2004-0884 15531524

[25] Zhang X Horwitz GA Heaney AP Nakashima M Prezant TR Bronstein MD Pituitary tumor transforming gene (PTTG) expression in pituitary adenomas. J Clin Endocrinol Metab (1999) 84:761–7. 10022450 10.1210/jcem.84.2.5432

[26] Pei L Melmed S Isolation and characterization of a pituitary tumor-transforming gene (PTTG). Mol Endocrinol (1997) 11:433–41. 9092795 10.1210/mend.11.4.9911

[27] Ishikawa H Heaney AP Yu R Horwitz GA Melmed S Human pituitary tumor-transforming gene induces angiogenesis. J Clin Endocrinol Metab (2001) 86:867–74. 10.1210/jcem.86.2.7184 11158059

[28] McCabe CJ Khaira JS Boelaert K Heaney AP Tannahill LA Hussain S Expression of pituitary tumour transforming gene (PTTG) and fibroblast growth factor-2 (FGF-2) in human pituitary adenomas: relationships to clinical tumour behaviour. Clin Endocrinol (Oxf) (2003) 58:141–50. 10.1046/j.1365-2265.2003.01598.x 12580928

[29] Saeger W Proliferation markers and cell cycle inhibitors in pituitary adenomas. Front Horm Res (2004) 32:110–26. 10.1159/000079040 15281342

[30] Mete O Lopes MB Overview of the 2017 WHO Classification of Pituitary Tumors. Endocr Pathol (2017) 28:228–43. 10.1007/s12022-017-9498-z 28766057

[31] Salehi F Agur A Scheithauer BW Kovacs K Lloyd RV Cusimano M Ki-67 in pituitary neoplasms: a review – part I. Neurosurgery (2009) 65:429–37. 10.1227/01.NEU.0000349930.66434.82 19687686

[32] Thapar K Kovacs K Scheithauer BW Stefaneanu L Horvath E Pernicone PJ Proliferative activity and invasiveness among pituitary adenomas and carcinomas: an analysis using the MIB-1 antibody. Neurosurgery (1996) 38:99–106. 10.1097/00006123-199601000-00024 8747957

[33] Pizarro CB Oliveira MC Coutinho LB Ferreira NP Measurement of Ki-67 antigen in 159 pituitary adenomas using the MIB-1 monoclonal antibody. Braz J Med Biol Res (2004) 37:235–43. 10.1590/S0100-879X2004000200011 14762579

[34] Mahta A Haghpanah V Lashkari A Heshmat R Larijani B Tavangar SM Non-functioning pituitary adenoma: immunohistochemical analysis of 85 cases. Folia Neuropathol (2007) 45:72–7. 17594597

[35] Smith TG Robbins PA Ratcliffe PJ The human side of hypoxia-inducible factor. Br J Haematol (2008) 141:325–34. 10.1111/j.1365-2141.2008.07029.x 18410568 PMC2408651

[36] Krock BL Skuli N Simon MC Hypoxia-induced angiogenesis: good and evil. Genes Cancer (2011) 2:1117–33. 10.1177/1947601911423654 22866203 PMC3411127

[37] Xiao Z Liu Q Zhao B Wu J Lei T Hypoxia induces hemorrhagic transformation in pituitary adenomas via the HIF-1alpha signaling pathway. Oncol Rep (2011) 26:1457–64. 10.3892/or.2011.1416 21822544

[38] Shan B Gerez J Haedo M Fuertes M Theodoropoulou M Buchfelder M RSUME is implicated in HIF-1-induced VEGF-A production in pituitary tumour cells. Endocr Relat Cancer (2012) 19:13–27. 10.1530/ERC-11-0211 22009797

[39] Li S Zhang Z Xue J Guo X Liang S Liu A Effect of hypoxia on DDR1 expression in pituitary adenomas. Med Sci Monit (2015) 21:2433–8. 10.12659/MSM.894205 26286316 PMC4547544

[40] Lee CZ Xue Z Zhu Y Yang GY Young WL Matrix metalloproteinase-9 inhibition attenuates vascular endothelial growth factor-induced intracerebral hemorrhage. Stroke (2007) 38:2563–8. 10.1161/STROKEAHA.106.481515 17673717

[41] Reyes R Guo M Swann K Shetgeri SU Sprague SM Jimenez DF Role of tumor necrosis factor-alpha and matrix metalloproteinase-9 in blood-brain barrier disruption after peripheral thermal injury in rats. J Neurosurg (2009) 110:1218–26. 10.3171/2008.8.JNS08382 19199470

[42] McColl BW Rose N Robson FH Rothwell NJ Lawrence CB Increased brain microvascular MMP-9 and incidence of haemorrhagic transformation in obese mice after experimental stroke. J Cereb Blood Flow Metab (2010) 30:267–72. 10.1038/jcbfm.2009.217 19826431 PMC2949124

[43] Mou C Han T Zhao H Wang S Qu Y Clinical features and immunohistochemical changes of pituitary apoplexy. J Clin Neurosci (2009) 16:64–8. 10.1016/j.jocn.2008.02.012 19046883

[44] Chen Z Li Z Chang Y Ma L Xu W Li M Relationship between NF-kappaB, MMP-9, and MICA expression in pituitary adenomas reveals a new mechanism of pituitary adenomas immune escape. Neurosci Lett (2015) 597:77–83. 10.1016/j.neulet.2015.04.025 25921632

[45] Paez Pereda M Ledda MF Goldberg V Chervin A Carrizo G Molina H High levels of matrix metalloproteinases regulate proliferation and hormone secretion in pituitary cells. J Clin Endocrinol Metab (2000) 85:263–9. 10.1210/jcem.85.1.6248 10634397

[46] Gultekin GD Cabuk B Vural C Ceylan S Matrix metalloproteinase-9 and tissue inhibitor of matrix metalloproteinase-2: prognostic biological markers in invasive prolactinomas. J Clin Neurosci (2015) 22:1282–7. 10.1016/j.jocn.2015.02.021 26071077

[47] Tsuge M Yasui K Ichiyawa T Saito Y Nagaoka Y Yashiro M Increase of tumor necrosis factor-alpha in the blood induces early activation of matrix metalloproteinase-9 in the brain. Microbiol Immunol (2010) 54:417–24. 10.1111/j.1348-0421.2010.00226.x 20618688

[48] Ryuto M Ono M Izumi H Yoshida S Weich HA Kohno K Induction of vascular endothelial growth factor by tumor necrosis factor alpha in human glioma cells. Possible roles of SP-1. J Biol Chem (1996) 271:28220–8. 8910439 10.1074/jbc.271.45.28220

[49] Polavarapu R Gongora MC Winkles JA Yepes M Tumor necrosis factor-like weak inducer of apoptosis increases the permeability of the neurovascular unit through nuclear factor-kappa B pathway activation. J Neurosci (2005) 25:10094–100. 10.1523/JNEUROSCI.3382-05.2005 16267216 PMC6725778

[50] Jayaraman T Paget A Shin YS Li X Mayer J Chaudhry H TNF-alpha-mediated inflammation in cerebral aneurysms: a potential link to growth and rupture. Vasc Health Risk Manag (2008) 4:805–17. 10.2147/VHRM.S2700 19065997 PMC2597764

[51] Mori K Tani M Kamata K Kawamura H Urata Y Goto S Mitogen-activated protein kinase, ERK1/2, is essential for the induction of vascular endothelial growth factor by ionizing radiation mediated by activator protein-1 in human glioblastoma cells. Free Radic Res (2000) 33:157–66. 10.1080/10715760000300711 10885623

